# Gelatin-Based Materials in Ocular Tissue Engineering

**DOI:** 10.3390/ma7043106

**Published:** 2014-04-17

**Authors:** James B. Rose, Settimio Pacelli, Alicia J. El Haj, Harminder S. Dua, Andrew Hopkinson, Lisa J. White, Felicity R. A. J. Rose

**Affiliations:** 1 School of Pharmacy, University of Nottingham, Nottingham NG7 2RD, UK; E-Mails: paxjr@nottingham.ac.uk (J.B.R.); lisa.white@nottingham.ac.uk (L.J.W.); 2 Department of Drug Chemistry and Technologies, “Sapienza” University of Rome, Piazzale Aldo Moro 5, 00185 Rome, Italy; E-Mail: settimio.pacelli@uniroma1.it; 3 Institute for Science and Technology in Medicine, Keele University, Stoke-on-Trent ST4 7QB, UK; E-Mail: a.j.el.haj@keele.ac.uk; 4 Academic Ophthalmology, Division of Clinical Neuroscience, University of Nottingham, Nottingham NG7 2RD, UK; E-Mails: harminder.dua@nottingham.ac.uk (H.S.D.); andrew.hopkinson@nottingham.ac.uk (A.H.)

**Keywords:** gelatin, tissue engineering, ophthalmology, biocompatibility, cornea, retinal epithelium

## Abstract

Gelatin has been used for many years in pharmaceutical formulation, cell culture and tissue engineering on account of its excellent biocompatibility, ease of processing and availability at low cost. Over the last decade gelatin has been extensively evaluated for numerous ocular applications serving as cell-sheet carriers, bio-adhesives and bio-artificial grafts. These different applications naturally have diverse physical, chemical and biological requirements and this has prompted research into the modification of gelatin and its derivatives. The crosslinking of gelatin alone or in combination with natural or synthetic biopolymers has produced a variety of scaffolds that could be suitable for ocular applications. This review focuses on methods to crosslink gelatin-based materials and how the resulting materials have been applied in ocular tissue engineering. Critical discussion of recent innovations in tissue engineering and regenerative medicine will highlight future opportunities for gelatin-based materials in ophthalmology.

## 1. Introduction

The emergence of regenerative medicine therapies in ophthalmology has provided potential treatment pathways for conditions which previously were untreatable [1–4]. One of the major challenges faced by those working in the area has been to develop cytocompatible, surgically deliverable scaffolds with good optical properties, which can be consistently produced at low cost. In the case of cell therapies, an additional requirement is that these scaffolds must reproducibly support sensitive populations of therapeutic cells [5,6]. Recent research has focused upon natural polymer proteins due to their similarity to native tissues [7–9].

Gelatin, a protein based material derived from the hydrolysis of collagen, has been well utilised in this area on account of its biodegradable, biocompatible nature and its commercial availability at low cost [10]. It has been shown to have advantages over its parent protein, which include lower immunogenicity [11–14], better solubility in aqueous systems and a sol-gel transition at 30 °C [15]. In addition, gelatin can be crosslinked or modified with the inclusion of other materials to significantly alter its mechanical and biochemical properties.

Research into the use of crosslinked gelatin-based materials in ocular repair has occurred in three distinct areas: bio-adhesives [16,17], structural scaffolds [18,19] and cell-sheet carriers [20,21] with an extensive body of research focused on the latter.

### 1.1. Gelatin as a Biomaterial

Gelatin exists as a mixture of water soluble protein fragments, comprised of the same amino acid sequences as collagen, from which it is derived [22]. Collagen differs from gelatin in that it contains far more tertiary structure, leading to a lower aqueous solubility [23]. Gelatin can be manufactured from a variety of animal collagens with the most commonly used forms in tissue engineering derived from porcine [24,25], fish [26] and bovine [27] tissues. Gelatin output quality can be dependent on the pH, temperature, and extraction time used in collagen processing [28]. Parameters such as molecular weight and isoelectric point (IEP) can be changed depending on the processing conditions [27,29]. Gelatin can be obtained under acidic and alkaline pre-treatment conditions which give rise to type A gelatin (IEP at pH 8–9) and type B gelatin (IEP at pH 4–5) respectively [28].

The Bloom strength of gelatin is a measure of gelation properties of the material and will largely be dictated by the triple-helix content [30–33]. Specifically the Bloom strength refers to the number of grams required for a 0.5 inch diameter probe to deflect a set gel by 4 mm, typically ranging from 30 to 300 g. It has been proposed that Bloom strength is related to the proline-hydroxyproline content of the resultant gelatin [34], which in turn will be determined by the composition of collagen being processed [28]. Typically fish gelatins possess lower levels of these important amino acids resulting in gelatin with lower gelling temperatures and lower bloom strength than those derived from mammals [35]. When using gelatin of porcine or gelatin source, religious beliefs (e.g., Islam, Judaism, Jainism and Hinduism) should be acknowledged. However, despite this mammalian gelatin has found wide use as an excipient in pharmaceutical processing [36], as a stabilizer in vaccines [37], and as an agent in food processing for many years [28].

The common use of gelatin in these applications has been shown in a very small number of cases to raise hypersensitivity to the protein [37,38]. However, reports of clinical reactivity to porcine and bovine gelatin have been relatively uncommon [39–41]. Immune reactivity has been reported to vary with molecular weight of gelatin being employed [42,43]. Thus considering the mode of processing and the specification of gelatin used will be important in clinical translation of tissue engineered constructs.

Mammalian gelatin, is rich in domains that bind to cell-surface receptors and to other extracellular matrix (ECM) proteins, such as fibronectin, offering an excellent substrate for attachment of adherent cells [44]. In addition, gelatin matrices can undergo collagenase mediated digestion, which allows biologically driven remodeling of the matrices *in vivo* [18]. Gelatin-based materials often have good transparency given their high water-content making them ideal for use in repair of ocular tissues in the visual axis [45].

### 1.2. Gelatin vs. Collagen

Collagen has been shown to be a suitable material for multiple applications in ophthalmology [7,46–49] however it is not without drawbacks. The main disadvantage of collagen is the potential antigenic and immunogenic response that can be elicited from its *in vivo* use [50]. Antigenicity of exogenous collagen is attributable both to the helical structures as well as central and terminal amino acid sequences. Gelatin has been reported to reduce the potential of an antigenic response *in vivo*, relative to its parent protein. Unlike collagen, gelatin is deficient in both tyrosine and tryptophan, and contains only low levels of phenylalanine [51]. Consequently, gelatin has a lower potential for the formation of aromatic radicals which have been linked to increased antigenic responses [52].

Issues with collagen’s immunogenic response have been circumvented through the use of recombinant human collagens (RHC), although these are costly to produce [53,54]. Chemically crosslinked RHC hydrogels have been used successfully to treat patients suffering corneal thinning [55]. Transplanted RHC constructs remained stable for 24 months post-surgery without the need for systemic immunosuppression [56].

The benefits of lower immunogenicity, cost and aqueous solubility make gelatin an excellent choice of base biomaterial for ocular tissue engineering applications. This review will take stock of the variety of different ways gelatin has been crosslinked and processed to create biocompatible scaffolds for ocular tissue engineering.

## 2. Crosslinking Strategies

Both gelatin and collagen have some solubility in aqueous systems, and can be rapidly digested by collagenases produced by many different cell types, both *in vivo* and *in vitro*. This is a significant drawback in terms of their utility in tissue engineering. To improve the physical properties of these proteins, crosslinking strategies have been developed to obtain materials with lower aqueous solubility, higher mechanical strength and stability against enzymatic degradation [51]. It has also been reported that crosslinking can significantly reduce the antigenicity of these materials, through significantly modifying major antigenic sites [57]. However, more recent accounts have reported that transplantation of xenogeneic glutaraldehyde crosslinked heart valves produced an increased antigenic response relative to uncrosslinked valves [58]. Currently, without substantiated evidence, it is difficult to gauge the *in vivo* effect of crosslinking on immunochemical responses to gelatin and collagen. For a review of collagen crosslinking processes readers are directed to an excellent review by Parenteau-Bareil *et al.* [59].

Crosslinking methods reported in the literature have included: chemical crosslinking; enzymatic crosslinking; and physical crosslinking. For an overview of the different crosslinking approaches that have been utilised for gelatin in ocular tissue engineering see Table 1.

### 2.1. Chemical Crosslinking

To date, chemical crosslinking is the most popular approach to crosslink gelatin. Chemical crosslinking agents can be differentiated into “non-zero-length” (covalently linking amine residues) and “zero-length” (covalently linking carboxylic acid and amine residues), depending on whether the linking reagent contributes to the molecular structure of the crosslinked product.

#### 2.1.1. Non-Zero-Length Crosslinking

Non-zero-length crosslinking introduces reagents to bind the gelatin network structure, typically by bridging the free amine groups of lysine or the free carboxylic acid residues of aspartic and glutamic acid in the gelatin structure. Several crosslinking reagents have been used to this end, including aldehydes (such as glyceraldehyde [60], formaldehyde [32] and glutaraldehyde (GA) [61]), polyepoxides [62], isocyanates [63] and natural products such as genipin [64,65].

GA has been used frequently on account of its fast reactions and high solubility in aqueous solution [60,66]. GA reacts with the α-amino groups of lysine to create a Schiff base between the polymer chains. GA crosslinking has for many years been the gold-standard for crosslinking porcine tissue derived heart valves for transplantation. Such devices have seen relatively low incidence of thromboembolism and good haemodynamic performance [67]. In comparison to crosslinking with other aldehydes, such as formaldehyde [32] and glyceraldehyde [60], the crosslinks made using GA are highly stable [68]. Crosslinking in this way can dramatically alter the mechanical and biological properties of gelatin as demonstrated in a study carried out on films using different concentrations of GA [66]. GA crosslinked gelatin has been investigated in ocular tissue engineering, with compatibility evaluated using corneal endothelial and stromal cells (see Table 1) [12,69].

Whilst GA crosslinked structures have been used clinically [67], constructs have caused inflammation and calcification when implanted *in vivo* in rabbit osteochondral defects [57,76]. This has prompted investigation of alternative crosslinkers. One non-zero-length crosslinker of common interest is genipin [64], a natural product abundantly present in gardenia fruits, which has been shown to be 10,000 times less toxic than GA [77]. The reaction between gelatin and genipin is not well characterized but it has been proposed to occur in two distinct steps (Scheme I). In the first step (Scheme IA) rapid nucleophilic attack of a lysine amino group to the ring structure of genipin results in the opening of the dihydropyran ring and the formation of a tertiary amine. A subsequent slower reaction (Scheme IB) then results in the crosslinking process with nucleophilic substitution by a lysine amino group from a second fragment of gelatin [65]. These two independent reactions lead to the crosslinking of gelatin, a reaction which is slower than that of GA. Whilst genipin has been used in ocular research to crosslink chitosan in corneal tissue engineering, no studies investigating genipin crosslinked gelatin were identified in the field of ocular tissue engineering [78,79].

#### 2.1.2. Zero-Length Crosslinking

One of the main disadvantages of non-zero-length crosslinking is that reagents built into the biomaterial are released upon degradation, which can potentially be a source of cell toxicity [57]. Zero-length crosslinker reagents typically activate carboxylic acid groups and facilitate their reaction with amine residues, resulting in the formation of an amide bond (Scheme II). The most common methods involve the use of acyl azide [80,81] and carbodiimide coupling [82]. These methodologies have been well developed in recent years although carbodiimide has been more widely employed than acyl azide coupling due to the toxicity of the hydrazine by-products [82].

One of the most well-used carbodiimide linkers is 1-ethyl-3-(3-dimethyl aminopropyl) carbodiimide (EDC) which activates the carboxylic acid residues of aspartic and glutamic acids and converts them into O-acylisourea groups (Scheme II). Amide bonds can be formed by nucleophilic attack of free amine groups of lysine on the activated carboxylic acid, with a urea derivative as a leaving group. Possible side-reactions occur through the hydrolysis of the O-acylisourea group or the rearrangement of the O-acylisourea group into a stable N-acylurea derivate [82]. N-hydroxysuccinimide (NHS) can be used in combination with EDC, to activate the carboxylic acid group, which is in turn less susceptible to hydrolysis, and can increase the efficiency of the crosslinking reaction. An advantage of this technique is that all residues are water soluble, and can be easily washed out of the construct after crosslinking. This crosslinking approach has been successfully applied to RHC to produce robust bio-artificial corneal grafts, that have been evaluated in a phase I clinical trial [56].

A recent study comparing the biocompatibility of EDC crosslinking to that of GA, demonstrated that EDC treated gels produced lower interleukin-1β and tumour necrosis factor-α expression in iris pigment epithelial cultures, as well as higher cell viability after 2 days (see Table 1) [12,71]. The EDC treated gels were seen to be safe and did not elicit any adverse events after 12 weeks of implantation in the anterior chamber of a rabbit eye [12]. The effect of solvent composition on the EDC crosslinking process has also been investigated. Increasing the ethanol content of solvent mixtures increased the crosslinking efficiency and did not elicit any significant increase in interleukin-6 (IL6) expression or drop in proliferative capacity of retinal epithelial cells [21].

### 2.2. Enzymatic Crosslinking

Recently, enzymes have been exploited to crosslink gelatin [16,17]. Calcium independent microbial transglutaminase catalyzes the formation of an amide bond between the carboxylic acid groups of glutamic acid and the ε-amino group of lysine [83]. This methodology has been used to produce bioadhesives suitable for treating retinal detachment [16]. Gelatin and microbial transglutaminase (mTG) were injected into the vitreous cavity of a rat model without the material eliciting structural or cellular damage to the retina. In addition, the gelatin-mTG adhesive was able to bind to bovine retinal tissue under wet conditions with lap-shear strengths comparable to other soft-tissue adhesives [16]. More recently gelatin-mTG adhesives have been evaluated for use in treating retinal tears in a rabbit model [17]. Gelatin-mTG complexes were seen to continue to adhere and seal retinal tears several days after administration with complete reattachment of the retina and without any inflammatory reactions. Other enzymes, such as tyrosinase, have also been evaluated in terms of gelatin crosslinking but have been found to form weaker gels [84].

### 2.3. Physical Crosslinking

Physical crosslinking methods of gelatin have been based on the use of plasma [85], UV radiation [86,87] and dehydrothermal treatment (DHT) [45,88]. Whilst physical crosslinking has advantages in that no potentially cytotoxic chemicals are introduced to the system, a lack of control over the reaction kinetics of crosslinking, as well as lower degrees of crosslinking in this method may produce mechanically weaker constructs [80,88] compared to chemical or enzymatic crosslinking. Although not strictly crosslinking, several studies have looked to utilize gelatin carriers formed from dehydration of gelatin solutions [20,73,75]. Low modulus constructs made in this way [20,75] are well suited to applications requiring rapidly resorbed cell sheet carriers for ocular delivery (see Table 1).

Both the IEP and molecular weight of gelatin have been investigated to evaluate the biocompatibility of dehydrated gelatin discs as carriers for corneal endothelial cells [29]. Gelatin with IEPs of 5.0 to 9.0 and several molecular weights in the range of 3 to 100 kDa were used to cast hydrogels disks which were subsequently dehydrated. The physiochemical and biocompatible properties of the transparent gels were assessed and the optimal gelatin disc carrier system was identified: gelatin with an IEP of 5.0 and molecular weight of 100 kDa. At the same molecular weight, gelatin with an IEP of 9.0 showed lower corneal endothelial compatibility suggesting that favorable cellular interactions occurred with gelatin that was negatively charged at physiological pH [29].

Whilst dehydrated gels with higher Bloom index can offer better mechanical properties in terms of mechanical strength and dissolution rate, they were seen to be less cell compatible when supplemented to the culture of retinal pigment epithelial (RPE) cells [30,31]. Higher Bloom gelatin discs induced higher levels of IL-6 and lower rates of proliferation of RPE cells suggesting that a higher Bloom index may increase cellular inflammatory reactions and reduce cell compatibility [31]. Investigations into sub-retinal RPE delivery using gelatin discs focused primarily on cannula delivery. It was noted that constructs made with low Bloom gelatin were too fragile to be delivered *in vivo* through a cannula into the sub-retinal space of a rabbit eye [30].

### 2.4. Chemical Modification of Gelatin

Outside the field of ocular tissue engineering, alternative strategies have been devised to enhance the level of control and the biocompatibility of the crosslinking process. One such method has been to chemically modify gelatin to give a starting prepolymer that can be UV crosslinked, affording constructs with mechanical properties that can be easily modulated [89]. The most common method to achieve this has been to use methacrylic anhydride to functionalise free amino groups of lysine as methacrylamide groups (Scheme III). Through dissolution in aqueous media with catalytic levels of photoinitiator, gelatin methacrylamide can be crosslinked with UV light [89,90].

The mechanical properties of crosslinked constructs have been changed through tailoring the level of methacrylation during gelatin methacrylamide synthesis [89]. Further control over the physical properties has also been attained through changing parameters such as: the concentration of gelatin methacrylamide and photoinitiator; and the UV irradiance delivered to the construct [91]. In this system, relatively low levels of UV exposure are able to produce stable hydrogels, which has allowed cells to be encapsulated and cultured within gelatin methacrylamide gels [92,93]. Exposures typically of around 7 mW.cm^−1^ have been used for less than 1 min. to generate cell-laden hydrogels with no reports of any significant effect on cell viability [89,93,94]. UV crosslinking of gelatin methacrylamide also offers opportunities for photopatterning allowing micro-features to be included into crosslinked constructs, through the use of photomasks and molds [94–96].

Applying gelatin methacrylamide gels to clinical biology could offer an opportunity to explore the effects of both matrix compliance and topographical cues on cell phenotype. In addition, UV crosslinking gelatin methacrylamide can form relatively transparent resultant hydrogels. These could offer real value in ocular tissue engineering, especially in producing 3D tissues such as the corneal stroma. Although gelatin methacrylamide has yet to be investigated in the field of ocular tissue engineering, the authors consider this to be an area of future consideration.

## 3. Tailoring Gelatin-Based Materials to Ocular Applications

**Figure 1.**
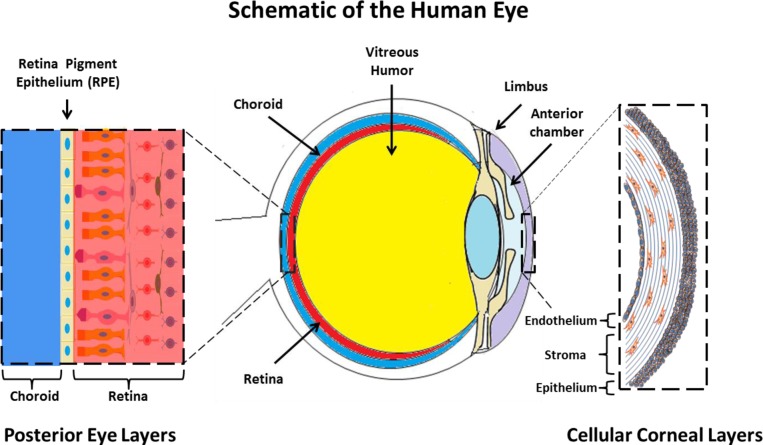
Schematic representation of the eye; gelatin-based materials have found application in the repair of the ocular components shown above.

Crosslinked gelatin biomaterials have found application in many ocular tissues: as a bio-adhesive to secure and stabilize retinal tissue [16,17], as a cell-sheet carrier for corneal endothelial cells [74], and as cellularised scaffolds for repair and regeneration of the corneal stroma (Figure 1) [19]. At the time of writing, only two relevant gelatin products could be identified, both used in bio-surgery: Gelfilm^®^, an absorbable film marketed for use in thoracic, ophthalmic and neuro- surgery [97] and Gelfoam^®^, a compressed gelatin sponge, marketed as a hemostatic device [98]. Current endeavors to develop gelatin-based biomaterials for regenerative medicine applications in ophthalmology are reported herein.

### 3.1. Cornea

The cornea exists as the multi-layered, transparent window covering the front of the eye [99]. The cornea is made up of six component layers: Epithelium; Bowman’s layer; Stroma; Descemet’s membrane; the newly described Dua’s layer and Endothelium [100]. Only the epithelium, stroma and endothelium are made up of cells, the structures of these three layers are shown in Figure 1. The cornea is responsible for a large proportion of the eyes’ refractive power, and thus its transparency is critical for normal function. A healthy cornea is avascular and immune privileged [101]. However in cases of trauma, burns and infectious disease, often vascularization and inflammation can occur which result in a loss of the transparency and immune privilege of the tissue [102].

Tissue engineered corneal tissues provide an alternative for patients suffering transplant rejection and also supplements the donor pool of corneal tissues [103]. The technology which has progressed the furthest is arguably that of the acellular recombinant human collagen scaffold which has had some success in clinical treatment of patients with corneal thinning [56,104]. In addition, scaffolds comprised of compressed collagen [8,48,49], amniotic membrane [105] and even decellularised corneas are currently under investigation [106].

Research into gelatin-based biomaterials for cornea tissue engineering is still preclinical and has mainly focused on providing substrates for cultivation and delivery of different corneal cell types [13]. These include the growth and delivery of corneal endothelial cells [73,74,107], growth of epithelial or limbal cells for corneal surface delivery [13,108], and investigation into the use of gelatin as a stromal replacement [18,19,69,108].

In addition, studies have examined the application of topical drugs to the ocular surface via gelatin particles [26,109–111] or by contact lenses containing immobilized gelatin particles loaded with hydrophilic protein [112].

#### 3.1.1. Corneal and Limbal Epithelium

Loss of the limbal epithelial stem cell (LESC) population can occur through trauma, burns, infectious or genetic disease and it is often associated with pain, inflammation and impaired vision [48]. LESC transplantation is often the only option for patients and involves grafting LESCs from a healthy donor eye, usually on an amniotic membrane carrier [113]. Whilst the procedure has shown success, biological variability of the amniotic membrane has produced varied levels of graft survival [6]. The search for new carriers has led to investigation into crosslinked gelatin biomaterials to provide low cost and effective solutions to this clinical problem.

De la Mata and colleagues recently reported the use of a LESC carrier composed of chitosan and gelatin covalently bound through crosslinking with glutaraldehyde and subsequently reduced with sodium borohydride [13]. Introduction of chitosan was an attempt to mimic the glycosaminoglycan composition of the native limbus, thought to be important in maintaining the “stemness” of seeded LESCs. This work demonstrated that at an optimum ratio of chitosan to gelatin (20:80), LESCs maintained a more stem-like phenotype than those cultured on tissue culture plastic (TCP) [114].

Cationised gelatin films have been used as a therapeutic bandage loaded with epidermal growth factor (EGF) to enhance wound healing in epithelial scars in an *in vivo* rabbit model [109]. Although cationised gelatin stabilized and controlled the release of epidermal growth factor, the films themselves did not have potential for further translation since they were too weak to be sutured in the rabbit model and required fixation through use of a soft contact lens. Additionally, the combination of a low water content hydrogel and the oxygen impermeable contact lens led to hypoxic conditions on the corneal epithelium and issues with re-epithelialization [109].

Another approach has been to chemically crosslink gelatin, collagen and hyaluronic acid with EDC and NHS in different ratios [115]. Hyaluronic acid is an important component of the extracellular matrix, which has been found to facilitate the adhesion and proliferation of corneal cells [116,117]. Seeding epithelial cells upon films made of a 3:6:1 ratio of gelatin, collagen and hyaluronic acid respectively increased proliferation compared to TCP. The combination reported represents an interesting example of a complex crosslinked mixture with a high porosity allowing satisfactory diffusion of important small molecules.

#### 3.1.2. Corneal Stroma

The main challenges in tissue engineering the corneal stroma lie in fabricating a fibrous extracellular matrix, sparsely populated with an even distribution of quiescent keratocytes and nerve fibres, into a single, strong, perfectly transparent construct [69]. Such a target would require either encapsulation or surface seeding of keratocytes upon a scaffold which would be supportive of a quiescent phenotype [118].

**Figure 2.**
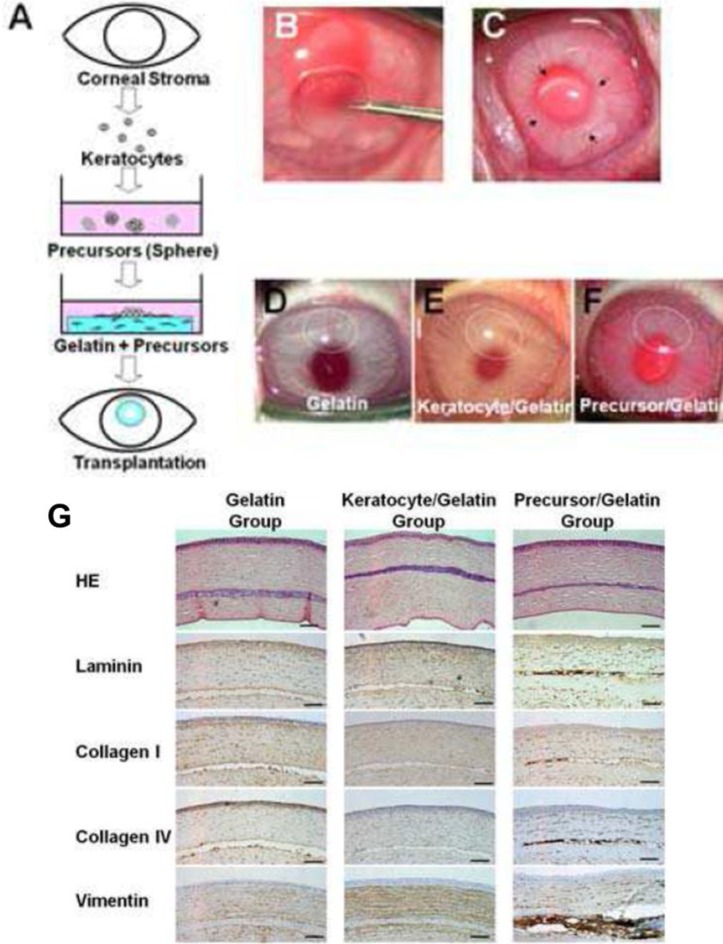

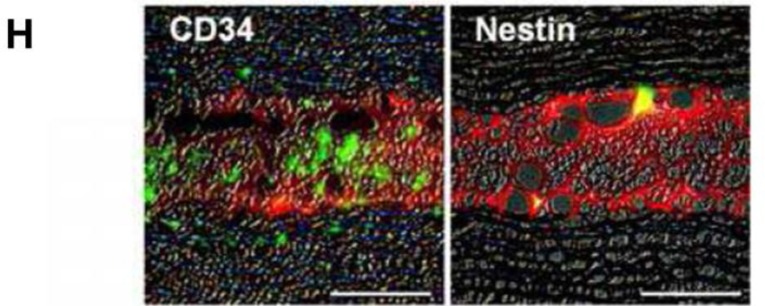
Assessment of the *in vivo* biocompatibility of cultured corneal stromal cells with GA crosslinked gelatin. (**A**) Culture of primary corneal stromal cell spheroids seeded upon a GA crosslinked gelatin hydrogel; (**B**,**C**) Implantation of construct within intra-stromal pockets in the rabbit cornea; (**D**,**E**,**F**) Visual appearance of constructs 4 weeks after implantation showing; (**D**) Gelatin; (**E**) Keratocyte and gelatin; and (**F**) Keratocyte precursor and gelatin; (**G**) Histological analysis of the implants 4 weeks after implantation showing no immune cell infiltration of the gelatin implants in any group. Keratocyte precursor cells showing more intense staining for laminin, type I and type IV collagen, and vimentin, Scale bar = 100 μm; (**H**) Immunolocalization of CD34 positive or nestin positive cells within the transplanted keratocytes precursor gelatin implants 4 weeks after transplantation. Rhodamine (red colour) shows the transplanted labelled corneal keratocyte precursors in the gelatin hydrogels, and FITC (green colour) shows the CD34- or nestin- positive cells. Scale bar = 100 μm. Courtesy of Mimura *et al.* [70] and Molecular Vision. Copyright by Mimura (2008). The work was originally published in [70].

Mimura *et al.* [70], attempted to circumvent difficulties in handling keratocytes by instead culturing keratocyte precursors. Stromal cells were isolated and cultured as spheroids within a GA crosslinked gelatin matrix (Figure 2A). This work demonstrated a novel method of reconstructing the corneal stroma: the keratocyte precursor spheres not only tolerated the GA crosslinked hydrogels but differentiated into mesenchymal fibroblasts and neural cells. Implantation into a rabbit corneal stromal pocket (Figure 2B–F) showed no immune cell infiltration even after 4 weeks of implantation, with keratocytes presenting desirable surface markers by histological immunostaining (Figure 2G–H) [69]. Although cell spheres required centrifugation after surface seeding to produce an even dispersion of the keratocyte precursors, this study demonstrated both *in vitro* and *in vivo* biocompatibility of GA crosslinked gelatin.

Hydrogels formed from covalently linking gelatin with hydroxypropyl chitosan have also been investigated for corneal stromal replacement [119], with a highly transparent and relatively permeable gel made to include chondroitin sulphate (CS), a linear anionic polysaccharide found in the corneal stroma [120]. It has been reported that introduction of CS improved gelatin hydrogel biocompatibility as seen by cell adhesion and proliferation data [18]. Lai *et al.* [18,19] have provided convincing evidence that incorporation of CS into gelatin hydrogels can enhance the biocompatibility of stromal replacement scaffolds. Firstly, the group demonstrated that increasing CS in the scaffold increased the total collagen and glycosaminoglycan (GAG) production by cultured rabbit corneal keratocytes without eliciting increased IL-6 expression [18]. However it was noted that the level of keratocan expression, an important biomarker of the keratocyte phenotype, was reduced in cells on scaffolds cultured on high CS content scaffolds. More recent studies have demonstrated that altering the crosslinking concentration (NHS-EDC) of gelatin–CS scaffolds significantly affected the fibronectin absorption, glucose permeability and keratocyte adhesion [19].

Yan and Gao [108,121] have reported investigations into the biocompatibility of gelatin-based electrospun membranes and the effect of fibre alignment upon keratocytes and epithelial cells. Keratocytes favoured aligned fibre scaffolds compared to random fibre orientations on gelatin-based scaffolds, and showed better proliferation [121] and keratocyte biomarker expression [108]. This work demonstrated that the use of scaffolds with instructive topographies, such as aligned electrospun scaffolds, could give rise to keratocytes expressing a more quiescent phenotype [122].

#### 3.1.3. Corneal Endothelium

The corneal endothelium covers the posterior surface of the cornea and plays a critical role in regulating the water content of the stroma. Conditions in which the endothelium is disrupted (e.g., Fuchs dystrophy) [123] often require surgical intervention to replace the endothelium, normally with a donor endothelium [124]. Given the limitations of donor corneal material, many researchers in this area have attempted to develop cultivated cell-sheet implants which could potentially be used to repair and regenerate the damaged endothelium [8]. For a detailed overview of this field, readers are directed to an excellent review by Mimura *et al.* [125].

A large body of work investigating endothelial cell-sheet delivery has focused on delivering cell sheets derived from thermoreversible poly-N-isopropylacrylamide (pNIPAM) culture substrates [107]. Following a small thermal change, polymeric culture surfaces release intact cell sheets ready for implantation at the posterior cornea [27]. One significant challenge faced by groups working in this area is the handling and transport of the cell sheet. To this end gelatin has been thoroughly investigated as a carrier substrate [20,73,74].

**Figure 3.**
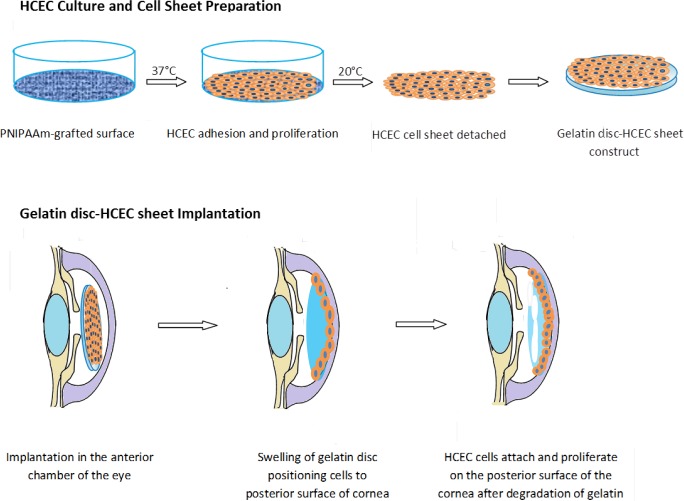
Schematic diagram of HCEC-gelatin sheet implantation reported by Lai *et al.* [73]. Primary endothelial cells were cultured upon a pNIPAM culture surface until confluent. The cell sheet was detached and transferred to a gelatin disc. The gelatin disc was implanted into the anterior chamber of the rabbit eye in which the endothelium had been removed surgically. The gelatin disc swelled localizing the corneal endothelial cells against the posterior surface of the cornea, where the cells proliferated and restored the cornea to health. Figure adapted from Hsiue *et al.* [74].

Lai *et al.* [73,107] were one of the first teams or groups to explore *in vivo* the possibility of an endothelialised gelatin carrier for endothelial sheet delivery to the posterior cornea. Culture of primary human corneal endothelial cells (HCECs) on poly-NIPAM substrates generated an intact endothelial cell sheet, which was transferred to a gelatin carrier. The resultant constructs were delivered to a de-endothelialised rabbit cornea *in vivo* and monitored for 6 months [73]. A schematic diagram of this process is presented in Figure 3. The use of cast hydrogels which were air-dried without crosslinking resulted in high swelling and dissolution rates of the gelatin discs, which rapidly dispersed in the anterior chamber, leaving no solid constructs in the visual axis. Investigations of intraocular pressure (IOP) and corneal thickness as measures of ocular health demonstrated that gelatin could be used effectively in this application. The transplanted HCEC-gelatin construct effectively restored both the IOP and corneal thickness close to that of the healthy cornea. In contrast, rabbit corneas receiving the gelatin carrier alone presented no reduction in corneal swelling, showing no signs of improved water regulation. This indicates that the cellular component really is important in restoring the function of the corneal endothelium *in vivo* [73]. In addition Lai *et al.* [29] showed that by using different molecular weights and isoelectric points of gelatin the mechanical and biocompatibility properties of air dried gelatin sheets could be changed.

Whilst this study was a good starting point for the use of gelatin hydrogels as a HCEC carrier, the use of the dense air dried gelatin scaffold was associated with reduced flow of aqueous humor which disrupted the flow of nutrients to other tissues and which could result in increased ocular pressure. To increase perfusion of aqueous humor, efforts have been made to increase the porosity of HCEC carrier membranes [72]. Highly porous membranes were achieved using a stirring-freeze drying process followed by chemical crosslinking. These porous gelatin films were seen to degrade in less than one day in physiological conditions; it was hypothesized that this would reduce swelling in the anterior chamber and hence improve nutrient perfusion.

Recently, it was further shown through a series of *in vivo* experiments that HCEC-gelatin constructs can be delivered to the target region effectively through a cannula. After delivery, corneal swelling occurring from de-endothelialisation was restored. The gelatin carriers dissolved rapidly within 2 weeks of implantation with no signs of biological reactions in the anterior chamber in test or control groups. These developments provide good evidence that this approach may be suitable for endothelial regeneration [27].

Other researchers have attempted to culture HCEC directly onto gelatin carrier films. Comparing dehydrothermally crosslinked gelatin sheets to atelocollagen, a water soluble form of collagen, the gelatin scaffolds were seen to offer better elasticity, transparency and permeability. However, in assessing the biocompatibility, the gelatin sheets were coated in collagen, making an accurate comparison between the two materials difficult [45].

Gelatin carrier sheets are able to play an important role in delivering endothelial cell sheets to the diseased corneal endothelium. This method has distinct advantages in ensuring there is no residual carrier material left in the anterior chamber, a challenge often faced when long lasting carriers such as amniotic membrane are employed [126].

### 3.2. Retinal Pigment Epithelium

The retinal pigment epithelium (RPE) is a highly specialized tissue, situated between the choroid and the neural retina. The tissue performs both metabolic and transport functions critical to maintaining the health of the neural retina [127]. These processes include transport of nutrients and waste products to and from photoreceptor cells [128]. Each cuboidal pigmented epithelium is estimated to support around 20 photoreceptors [129].

Dry age-related macular degeneration (AMD) is one of largest cause of blindness in developed nations. Prevalence of the disease continues to rise with the increase in average life expectancy. Loss of vision in dry AMD is the result of degeneration of photoreceptors occurring as RPE cells with which they are associated deteriorate and die. With the exception of macular translocation, there are currently no therapies available for the treatment of dry-AMD [1].

**Figure 4.**
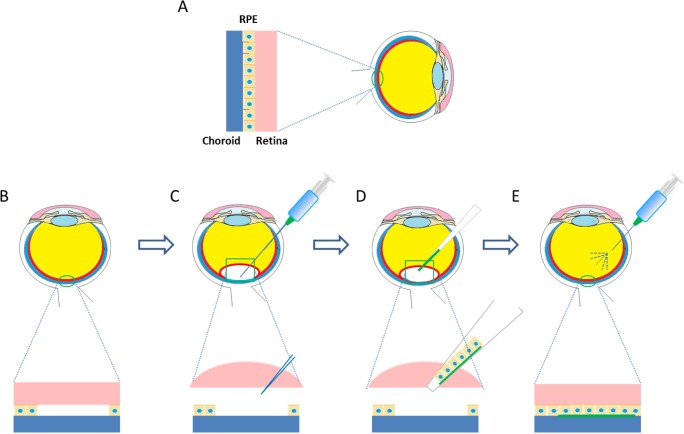
Potential treatment of RPE degeneration with RPE sheet-gelatin constructs. (**A**) Schematic of healthy eye, presenting an intact retinal pigment between the retina and the choroid; (**B**) degenerated RPE cell layer leading to potential loss of vision; (**C**) small portion of retina detached from RPE layer through introduction of fluid in the sub-retinal space; (**D**) cell sheet and carrier introduced into sub-retinal space through cannula delivery; (**E**) vitreous replacement fluids used to restore intraocular pressure and position detached portion of retina back in contact with implant.

One strategy currently being explored for the treatment of dry AMD, amongst other macular dystrophies is that of RPE transplantation. Such a therapy would involve the delivery of donor RPE cells, or allogeneic RPE progenitors beneath the neural retina, in order to restore the damaged tissue [75]. Whilst a bolus injection of RPE cells into the sub-retinal space has been demonstrated to be effective [130], issues with cellular positioning and cell survival after injection have been reported [131]. Strategies to overcome these hurdles have included the use of injectable cell laden hydrogels to prevent death of cells by reflux [132] or through generating RPE sheets [133] that can be transplanted either with [134] or without a carrier system [134,135]. A schematic of the transplantation of RPE-carrier sheets is presented in Figure 4.

There are several literature reports of the use of gelatin as an RPE sheet carrier which have offered signs of success. Hsiue *et al.* [75] reported initial success with a gelatin-cell construct in which sheets of confluent RPE cells were sandwiched between two gelatin discs. One study investigated an array of treatments for the sterilization of gelatin membrane carriers including: hydrogen peroxide gas plasma, ethylene oxide gas, and γ-ray irradiation, to assess their cytotoxicity towards RPE cells. Scaffolds sterilized with, low dose γ-ray irradiation (16.6 kGy), were seen to be least cytotoxic *in vitro* and well tolerated when injected into the sub-retinal space in a rabbit model, with no signs of inflammation. The effects of Bloom strength have also been assessed in the gelatin-RPE-gelatin sandwich. Lower Bloom gelatins were generally easier to deliver through a cannula to the sub-retinal space, and dissolved faster and were cytocompatible relative to higher Bloom gelatin [30]. Lai *et al.* [31] examined the cell compatibility of gelatin Bloom strengths and suggested that lower Bloom strengths were associated with lower levels of inflammation as measured by the production of the pro-inflammatory cytokine IL-6.

Both GA and EDC crosslinked gelatin membranes have been investigated for retinal epithelial sheet tissue engineering [71]. A comparison of the biocompatibility of the two crosslinkers demonstrated that EDC was less cytotoxic, as demonstrated by a proliferation assay. In addition ARPE-19 cells expressed higher levels of IL6 when cultured on GA crosslinked gelatin.

An evaluation of the effect of crosslinker concentration of EDC (0–0.4 mmol EDC/mg gelatin) on physical and biocompatibility properties showed that increased crosslinker concentrations decreased water solubility and collagenase digestion times. Higher concentrations of EDC (0.1–0.4 mmol) reduced the biocompatibility of the membranes as measured through an MTT assay. The effect of solvent composition was also investigated showing that the crosslinking density could be controlled using different ethanol to water ratios [21].

### 3.3. Bio-Adhesives for Retinal Tears

The investigation of gelatin-based materials as bio-adhesives for the treatment of retinal detachment has been of interest to a number of research groups in this area [136]. Studies have investigated the potential use of microbial transglutaminase (m-TG) crosslinked gelatin as a bio-adhesive [16,17]. Enzymatic crosslinking has been shown to be well suited to this application given the need for *in situ* gelling. Gelatin solutions 20% (w/v) were made up and mixed for 1 minute with a 20% (V/V) solution of microbial transglutaminase [16]. After injection of the bio-adhesive into the vitreous cavity of a rat model, there were no signs of any inflammatory or antigenic response after 2 weeks, demonstrating the *in vivo* biocompatibility of the adhesive. With a more concentrated solution of the adhesive, an *in vitro* lap shear assessment showed that a lap shear moduli of 12–25 kPa could be achieved, similar to other soft tissue adhesives (12–20 kPa) [16].

**Figure 5.**
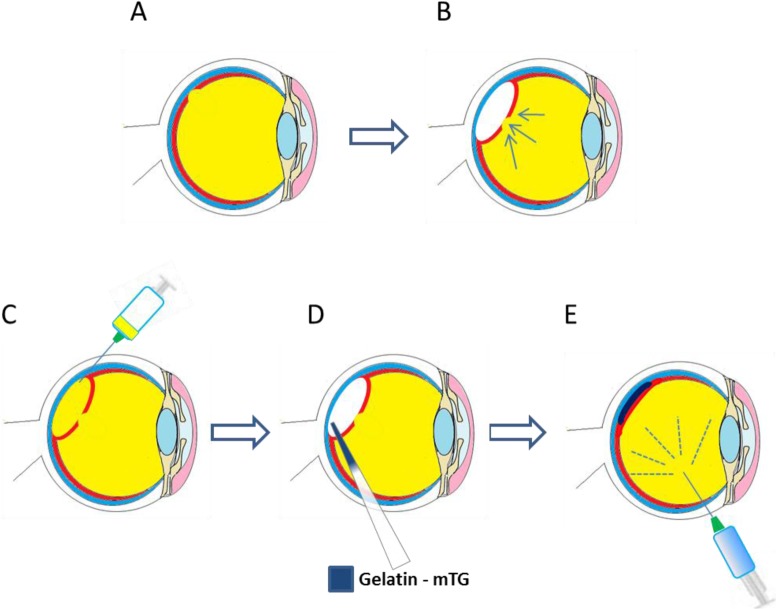
Potential treatments of retinal tears with m-TG. (**A**) Retinal tear initially forms; (**B**) if large enough vitreous humor diffuses into sub-retinal space exacerbating the tear and forcing more retinal tissue away from the RPE; (**C**) sub-retinal vitreous humor aspirated; (**D**) a homogenous mixture of gelatin and m-TG solutions mixed and applied to the sub-retinal space; (**E**) normal ocular pressure restored through infusion of vitreous replacement fluid.

Gelatin solutions 15% (w/v) with microbial transglutaminase have been used *in vivo* in a vitrectomy model (simulating retinal detachment) [137]. The bio-adhesive was seen to successfully cover a retinal tear model 7 days after administration. Optical coherence tomography demonstrated that the gelatin material had completely disappeared by day three. In addition, electroretinograms showed no adverse effects of the gelatin–mTG adhesive on retinal function. A schematic of how the bioadhesive could be used for the repair of retinal tears is shown in Figure 5.

## 4. Opportunities for Future Work

Gelatin-based materials have been most successful in ocular tissue engineering as cell sheet carriers, with effective delivery of both corneal endothelial sheets to the posterior cornea [20], and also RPE sheets to the sub-retinal space [138]. In both applications dehydrated gelatin discs were successfully delivered through a cannula and were swiftly resorbed *in vivo*, with no adverse biological events reported.

In addition to altering the bulk chemistry of gelatin, the process of crosslinking also changes both the matrix compliance and potentially the topography of the constructs [139]. The work described in this review largely attributes changes in biocompatibility to the toxicity and antigenicity of the chemical changes [12]. Future work to evaluate the effect of matrix elasticity of gelatin grafts is an important step to develop a deeper understanding of the biocompatibility of gelatin. Gelatin methacrylamide offers a gel-based system with tunable matrix stiffness, which can be controlled without significantly changing the chemical composition [95]. Generation of gelatin methacrylamide gels of different elastic moduli [89] has been previously demonstrated. In the authors opinion this material would be an effective tool in determining the optimum material properties for application in ocular tissue engineering.

Photocrosslinkable gelatin scaffolds in recent years have been a popular choice for use in a range of tissue engineering applications including cartilage repair [140], generating blood vessels [93] and also cardiac tissue development [141]. However to the authors’ knowledge this highly versatile material has yet to be explored in ocular tissue engineering. The authors hypothesize that the additional spatial control of crosslinking offered through photo- patterning [94,142], molding [141] and stereolithography [95] will provide a range of opportunities in both creating candidate scaffolds for stromal tissue engineering and also in exploring the influence of matrix compliance and topography on ocular cell types.

## 5. Summary

Crosslinked gelatin scaffolds make up a small but important part of the ocular tissue engineering landscape. Gelatin’s similarity to collagen offers an excellent, low cost starting substrate that if crosslinked using appropriate methods could provide lower antigenic and immunogenic risk than its parent material.

Gelatin and its derivatives have been used as potential scaffolds for corneal epithelium [13], corneal endothelium [20] and retinal pigment epithelium [21], as a bioartificial corneal stroma [19] and as a potential bioadhesive in treatment of retinal detachment [17]. The range of crosslinking options used to strengthen gelatin scaffolds in this field to date is extensive. There is good evidence to suggest that zero-length crosslinkers may be preferable in terms of both cell compatibility and biocompatibility [71]; the authors’ review of recent literature suggests this is a more popular method than non-zero-length crosslinking. Whilst there may be uses for glutaraldehyde to crosslink matrices, such as fragile electrospun matrices which are short lived in aqueous solutions [60], the risk of cell toxicity of excipients could be a potential issue.

The utility of dehydrated gelatin discs in ocular tissue engineering has been dominated by application as a cell sheet carrier in the delivery of either endothelial cell sheets to the posterior cornea [27], or retinal pigment epithelial cells to the sub-retinal space [30,75]. These gelatin carriers have provided effective cell delivery *in vivo* and have been well-tolerated with few adverse events reported [27].

Over the last decade the rise in popularity of partial thickness corneal transplants [123,143] has led to an opportunity to supplement the insufficient donor cornea pool with tissue engineered partial thickness grafts. To date there have been few applications of gelatin in this area and studies have examined cell toxicity [19] rather than creation of functional tissues. One issue that is likely to have limited research in this area is the difficulty in forming even dispersions of corneal stromal cells through gelatin matrices, with the harsh or lengthy crosslinking processes not amenable to producing cell laden constructs. Developments in other areas of tissue engineering have shown that photo crosslinkable gelatin products can be used to good effect to create cell-laden hydrogels [94,95]. The application of photo crosslinkable gelatin in corneal stromal tissue engineering is foreseen by the authors as being an area which must be investigated, particularly if gelatin-based materials are to fulfil their potential in stromal tissue engineering.

## Figures and Tables

**Scheme I.**
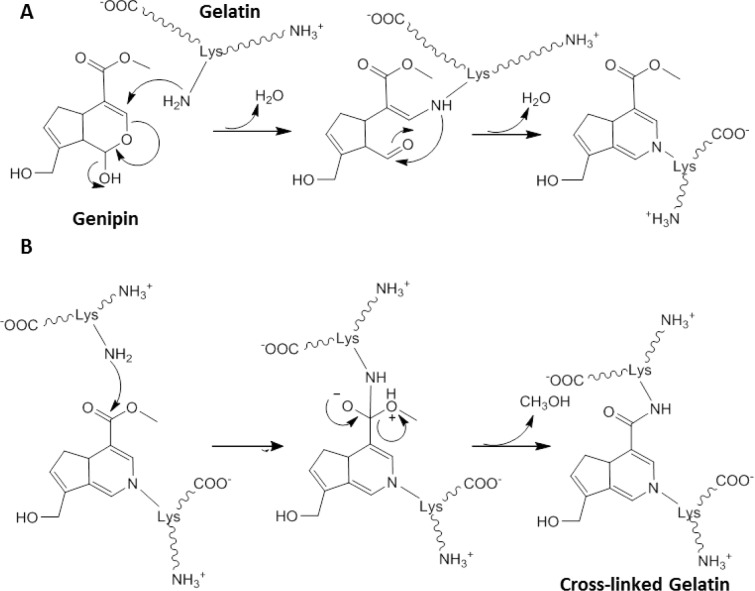
Crosslinking reaction of gelatin by genipin with: (**A**) primary reaction through Michael addition to form stable intermediate; and (**B**) secondary reaction with nucleophilic substitution of free lysine amine molecules into genipin activated ester.

**Scheme II.**
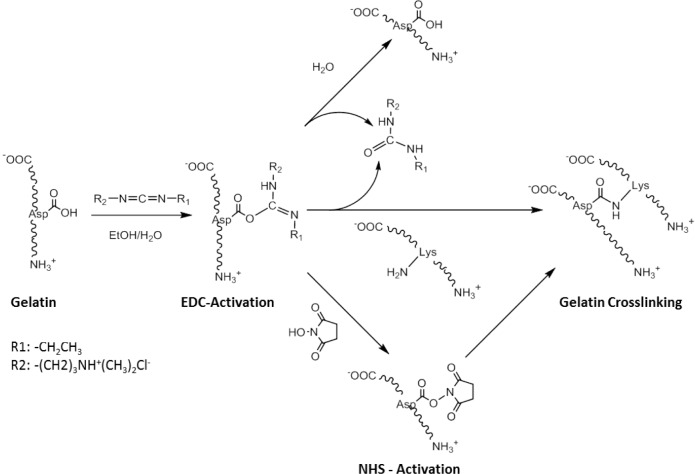
Schematic of the mechanism of the crosslinking reaction between carboxylic acids and lysine, through activation with 1-ethyl-3-(3-dimethyl-aminopropyl)carbodiimide (EDC) and N-hydroxysuccinamide (NHS). The amide bond is formed directly between the two amino acids of gelatin with no linker in between.

**Scheme III.**
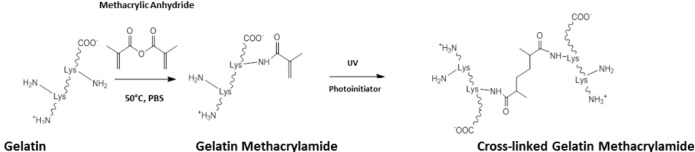
Methacrylation of gelatin to afford gelatin methacrylamide. Free amino groups of gelatin react with methacrylic anhydride in phosphate buffered saline solution (PBS) at 50 °C. The gelatin methacrylamide product can be photocrosslinked through the use of a photo activated radical initiator and UV light [89].

**Table 1.** Application of crosslinked gelatin in ocular tissue engineering.

Type of crosslinking		Gel composition	Cell type	Target tissue	Clinical application	Validation of biocompatibility		Reference
						*in vitro* experiments	*in vivo* experiments	
**Chemical: non-zero-length**	GA	10% Gelatin	Primary corneal endothelial	Corneal endothelium	Endothelial cell sheet delivery	Proliferation assay; live/dead; PIC expression	IOP; cell morphology corneal thickness;	[12]
	GA	10% Gelatin	Keratocyte sphere	Corneal stroma	Stromal tissue graft	ICC; gene expression; histology	Biomacroscopy; histology; IHC	[69,70]
	GA	Gelatin and Chitosan	Limbal epithelial cells	Limbal epithelium	Limbal Epithelial Stem Cell Carrier	Proliferation assay; gene expression; ICC	–	[13]
	GA	10% Gelatin	ARPE-19 Cells	RPE	RPE Sheet Delivery	Proliferation assay PIC expression	–	[71]

**Chemical: zero-length**	EDC	10% Gelatin	Primary Corneal Endothelial	Corneal Endothelium	–	Proliferation assay; Live/Dead; PIC expression	IOP; Corneal Thickness; Morphology; Biomacroscopy; h-Proline in aq. humor; Histology; IOP;	[12,72]
	EDC	10% Gelatin	ARPE-19 Cells	RPE	RPE Sheet Delivery	Proliferation; Live/Dead; PIC expression; glutamate uptake;	–	[21]
	EDC	15% Gelatin	Primary Corneal Endothelial	Corneal Endothelium	Endothelial Cell Sheet Delivery	Proliferation assay; Endothelial Gene Expression	Endothelial Cell Density	[20]
	EDC/NHS	10% Freeze dried crosslinked with CS	Primary Corneal Stromal Cells	Corneal Stroma	Stromal Tissue Graft	Adhesion assay; Proliferation assay; Collagen and GAG production; gene expression; Cell morphology	–	[18,19]

**Enzyme**	m-TG	22.5% Gelatin + 20mg/mL m-TG	–	Neural Retina	Retinal Reattachment	–	Histology	[16]
	m-TG	12% Gelatin/10.8 IU/mL m-TG	–	Neural Retina	Retinal Reattachment	–	Fundus Examination; Histology; OCT	[17]

**Physical**	Dehydration	10% Gelatin	Primary Corneal Endothelial	Corneal Endothelium	Endothelial Cell Sheet Delivery	ICC; Live/Dead; Morphology (SEM and TEM); Western Blot	Biomacroscopy; Corneal Thickness, Histology, Localization Studies	[27,29,73,74]
	Dehydration	10% Gelatin	Primary Rat IPE cells; ARPE19 cell line	RPE	RPE Sheet Delivery	Morphology; Proliferation assay; Live/Dead; Glutamate uptake; PIC expression	Graft survival Histology Post-Implantation	[30,31,75]
	DHT	Gelatin	Primary Corneal Endothelial	Corneal Endothelium	Endothelial Cell Sheet Delivery	ICC; Cell morphology	–	[45]

CS, chondroitin sulphate; DHT, dehydrothermal; GA, glutaraldehyde; GAG, glycosaminoglycan; ICC, immunocytochemistry; IHC, immunohistochemistry; IOP, intraocular pressure; IPE, iris pigment epithelial cells; m-TG, microbial transglutaminase; OCT, optical coherence tomography; PIC, pro-inflammatory cytokine; SEM, scanning electron microscopy; RPE, retinal pigment epithelial.

## References

[1] Carr A.-J.F., Smart M.J.K., Ramsden C.M., Powner M.B., da Cruz L., Coffey P.J. (2013). Development of human embryonic stem cell therapies for age-related macular degeneration. Trends Neurosci.

[2] Ahmad S., Kolli S., Lako M., Figueiredo F., Daniels J.T. (2010). Stem cell therapies for ocular surface disease. Drug Discov. Today.

[3] Baylis O., Figueiredo F., Henein C., Lako M., Ahmad S. (2011). 13 years of cultured limbal epithelial cell therapy: A review of the outcomes. J. Cell. Biochem.

[4] Pinnamaneni N., Funderburgh J.L. (2012). Stem cells in the corneal stroma. Stem Cells.

[5] Shortt A.J., Secker G.A., Lomas R.J., Wilshaw S.P., Kearney J.N., Tuft S.J., Daniels J.T. (2009). The effect of amniotic membrane preparation method on its ability to serve as a substrate for the *ex vivo* expansion of limbal epithelial cells. Biomaterials.

[6] Deshpande P., McKean R., Blackwood K.A., Senior R.A., Ogunbanjo A., Ryan A.J., MacNeil S. (2010). Using poly(lactide-co-glycolide) electrospun scaffolds to deliver cultured epithelial cells to the cornea. Regen. Med.

[7] Duan X., Sheardown H. (2006). Dendrimer crosslinked collagen as a corneal tissue engineering scaffold: Mechanical properties and corneal epithelial cell interactions. Biomaterials.

[8] Levis H.J., Peh G.S.L., Toh K.-P., Poh R., Shortt A.J., Drake R.A.L., Mehta J.S., Daniels J.T. (2012). Plastic compressed collagen as a novel carrier for expanded human corneal endothelial cells for transplantation. PLoS One.

[9] Fagerholm P., Lagali N.S., Carlsson D.J., Merrett K., Griffith M. (2009). Corneal regeneration following implantation of a biomimetic tissue-engineered substitute. Clin. Transl. Sci.

[10] Jones R.T., Podczeck F., Jones B.E. (2004). Gelatin: Manufacture and Physio-Chemical Properties. Pharmaceutical Capsules.

[11] Jain E., Kumar A. (2013). Disposable polymeric cryogel bioreactor matrix for therapeutic protein production. Nat. Protoc.

[12] Lai J.-Y. (2010). Biocompatibility of chemically cross-linked gelatin hydrogels for ophthalmic use. J. Mater. Sci. Mater. Med.

[13] De la Mata A., Nieto-Miguel T., López-Paniagua M., Galindo S., Aguilar M.R., García-Fernández L., Gonzalo S., Vázquez B., Román J.S., Corrales R.M. (2013). Chitosan-gelatin biopolymers as carrier substrata for limbal epithelial stem cells. J. Mater. Sci. Mater. Med.

[14] Tonsomboon K., Strange D.G.T., Oyen M.L. Gelatin Nanofiber-Reinforced Alginate Gel Scaffolds for Corneal Tissue Engineering.

[15] Bohidar H.B., Jena S.S. (1994). Study of sol-state properties of aqueous gelatin solutions. J. Chem. Phys.

[16] Chen T., Janjua R., McDermott M.K., Bernstein S.L., Steidl S.M., Payne G.F. (2006). Gelatin-based biomimetic tissue adhesive. Potential for retinal reattachment. J. Biomed. Mater. Res. B Appl. Biomater.

[17] Yamamoto S., Hirata A., Ishikawa S., Ohta K., Nakamura K., Okinami S. (2013). Feasibility of using gelatin-microbial transglutaminase complex to repair experimental retinal detachment in rabbit eyes. Graefe’s Arch. Clin. Exp. Ophthalmol.

[18] Lai J.-Y., Li Y.-T., Cho C.-H., Yu T.-C. (2012). Nanoscale modification of porous gelatin scaffolds with chondroitin sulfate for corneal stromal tissue engineering. Int. J. Nanomed.

[19] Lai J.-Y., Corneal Stromal, Cell Growth (2013). on Gelatin/Chondroitin Sulfate Scaffolds Modified at Different NHS/EDC Molar Ratios. Int. J. Mol. Sci.

[20] Lai J.-Y., Ma D.H.-K., Lai M.-H., Li Y.-T., Chang R.-J., Chen L.-M. (2013). Characterization of cross-linked porous gelatin carriers and their interaction with corneal endothelium: Biopolymer concentration effect. PLoS One.

[21] Lai J.-Y. (2013). Influence of solvent composition on the performance of carbodiimide cross-linked gelatin carriers for retinal sheet delivery. J. Mater. Sci. Mater. Med.

[22] Lou X., Chirila T. (1999). Swelling behaviour and mechanical properties of chemically cross-linked gelatin gels for biomedical use. J. Biomater. Appl.

[23] Pahuja P., Arora S., Pawar P. (2012). Ocular drug delivery system: A reference to natural polymers. Expert Opin. Drug Deliv.

[24] Natu M.V, Sardinha J.P., Correia I.J., Gil M.H. (2007). Controlled release gelatin hydrogels and lyophilisates with potential application as ocular inserts. Biomed. Mater.

[25] Lai J.-Y., Hsieh A.-C. (2012). A gelatin-g-poly(N-isopropylacrylamide) biodegradable *in situ* gelling delivery system for the intracameral administration of pilocarpine. Biomaterials.

[26] Jain D., Carvalho E., Banthia A.K., Banerjee R. (2011). Development of polyvinyl alcohol-gelatin membranes for antibiotic delivery in the eye. Drug Dev. Ind. Pharm.

[27] Hsu W.-M., Chen K.-H., Lai J.-Y., Hsiue G.-H. (2013). Transplantation of human corneal endothelial cells using functional biomaterials: Poly(*N*-isopropylacrylamide) and gelatin. J. Exp. Clin. Med.

[28] Gómez-Guillén M.C., Giménez B., López-Caballero M.E., Montero M.P. (2011). Functional and bioactive properties of collagen and gelatin from alternative sources: A review. Food Hydrocoll.

[29] Lai J.-Y., Lu P.-L., Chen K.-H., Tabata Y., Hsiue G.-H. (2006). Effect of charge and molecular weight on the functionality of gelatin carriers for corneal endothelial cell therapy. Biomacromolecules.

[30] Lai J., Lin P., Hsiue G., Cheng H., Huang S. (2009). Low bloom strength gelatin as a carrier for potential use in retinal sheet encapsulation and transplantation. Biomacromolecules.

[31] Lai J.-Y. (2009). The role of bloom index of gelatin on the interaction with retinal pigment epithelial cells. Int. J. Mol. Sci.

[32] Usta M., Piech D.L., MacCrone R.K., Hillig W.B. (2003). Behavior and properties of neat and filled gelatins. Biomaterials.

[33] Bigi A., Panzavolta S., Rubini K. (2004). Relationship between triple-helix content and mechanical properties of gelatin films. Biomaterials.

[34] Ledward D., Mitchell J., Ledward D. (1986). Gelation of gelatin. Functional Properties of Food Macromolecules.

[35] Farris S., Schaich K.M., Liu L., Piergiovanni L., Yam K.L. (2009). Development of polyion-complex hydrogels as an alternative approach for the production of bio-based polymers for food packaging applications: A review. Trends Food Sci. Technol.

[36] Djagny V.B., Wang Z., Xu S. (2001). Gelatin: A valuable protein for food and pharmaceutical industries: Review. Crit. Rev. Food Sci. Nutr.

[37] Sakaguchi M., Inouye S. (2000). IgE sensitization to gelatin: The probable role of gelatin-containing diphtheria–tetanus–acellular pertussis (DTaP). Vaccines.

[38] Marrel J., Christ D., Spahn D. (2011). Anaphylactic shock after sensitization to gelatin. Br. J. Anaesth.

[39] Mullins R.J. (2003). Anaphylaxis: Risk factors for recurrence. Clin. Exp. Allergy.

[40] Pool V., Braun M.M., Kelso J.M., Mootrey G., Chen R.T., John W., Jacobson R.M., Gargiullo P.M., Pool V., Braun M.M. (2002). Prevalence of anti-gelatin IgE antibodies in people with anaphylaxis after measles-mumps-rubella vaccine in the United States. Pediatrics.

[41] Sakaguchi M., Hori H., Hattori S., Irie S. (1999). IgE reactivity to α1 and α2 chains of bovine type I collagen in children with. J. Allergy Clin. Immunol.

[42] Kelso J.M. (1999). The gelatin story. J. Allergy Clin. Immunol.

[43] Sakai Y., Yamato R., Onuma M., Kikuta M., Watanabe M., Nakayama T. (1998). Non-antigenic and low allergic gelatin produced by specific digestion with an enzyme-coupled matrix. Biol. Pharm. Bull.

[44] Katagiri Y., Brew S.A., Ingham K.C. (2003). All six modules of the gelatin-binding domain of fibronectin are required for full affinity. J. Biol. Chem.

[45] Watanabe R., Hayashi R., Kimura Y., Tanaka Y., Nishida K. (2011). A novel gelatin hydrogel carrier sheet for corneal. Tissue Eng. Part. A.

[46] Ahn J.-I., Kuffova L., Merrett K., Mitra D., Forrester J.V., Li F., Griffith M. (2013). Crosslinked collagen hydrogels as corneal implants: Effects of sterically bulky *vs.* non-bulky carbodiimides as crosslinkers. Acta Biomater.

[47] Duan X., McLaughlin C., Griffith M., Sheardown H. (2007). Biofunctionalization of collagen for improved biological response: Scaffolds for corneal tissue engineering. Biomaterials.

[48] Levis H.J., Massie I., Dziasko M.A., Kaasi A., Daniels J.T. (2013). Rapid tissue engineering of biomimetic human corneal limbal crypts with 3D niche architecture. Biomaterials.

[49] Xiao X., Pan S., Liu X., Zhu X., Connon C.J., Wu J., Mi S. (2013). *In vivo* study of the biocompatibility of a novel compressed collagen hydrogel scaffold for artificial corneas. J. Biomed. Mater. Res.Part A.

[50] Lynn A.K., Yannas I.V., Bonfield W. (2004). Antigenicity and immunogenicity of collagen. J. Biomed. Mater. Res. Part B Appl. Biomater.

[51] Gorgieva S., Kokol V., Pignatello R. (2011). Collagen- *vs.* Gelatine-Based Biomaterials and Their Biocompatibility: Review and Perspectives. Biomaterials Applications for Nanomedicine.

[52] Kokare C.R., Prakashan N. (2008). Pharmaceutical Microbiology-Principles and Applications.

[53] Liu W., Merrett K., Griffith M., Fagerholm P., Dravida S., Heyne B., Scaiano J.C., Watsky M.A., Shinozaki N., Lagali N. (2008). Recombinant human collagen for tissue engineered corneal substitutes. Biomaterials.

[54] Olsen D. (2003). Recombinant collagen and gelatin for drug delivery. Adv. Drug Deliv. Rev.

[55] Merrett K., Fagerholm P., McLaughlin C.R., Dravida S., Lagali N., Shinozaki N., Watsky M.A., Munger R., Kato Y., Li F. (2008). Tissue-engineered recombinant human collagen-based corneal substitutes for implantation: Performance of type I *versus* type III collagen. Invest. Ophthalmol. Vis. Sci.

[56] Fagerholm P., Lagali N.S., Merrett K., Jackson W.B., Munger R., Liu Y., Polarek J.W., Söderqvist M., Griffith M. (2010). A biosynthetic alternative to human donor tissue for inducing corneal regeneration: 24-month follow-up of a phase 1 clinical study. Sci. Transl. Med.

[57] Speer D.P., Chvapil M., Eskelson C.D., Ulreich J. (1980). Biological effects of residual glutaraldehyde in glutaraldehyde-tanned collagen biomaterials. J. Biomed. Mater. Res.

[58] Courtman D.W., Errett B.F., Wilson G.J. (2001). The role of crosslinking in modification of the immune response elicited against xenogenic vascular acellular matrices. J. Biomed. Mater. Res.

[59] Parenteau-Bareil R., Gauvin R., Berthod F. (2010). Collagen-Based biomaterials for tissue engineering applications. Materials.

[60] Sisson K., Zhang C., Farach-Carson M.C., Chase D.B., Rabolt J.F. (2009). Evaluation of cross-linking methods for electrospun gelatin on cell growth and viability. Biomacromolecules.

[61] Talebian A., Kordestani S.S., Rashidi A., Dadashian F., Montazer M. (2007). The effect of glutaraldehyde on the properties of gelatin films. DOAJ.

[62] Nishi C., Nakajima N., Ikada Y. (1995). *In vitro* evaluation of cytotoxicity of diepoxy compounds used for biomaterial modification. J. Biomed. Mater. Res.

[63] Naimark W.A., Pereira C.A., Tsang K., Lee J.M. (1995). HMDC crosslinking of bovine pericardial tissue: A potential role of the solvent environment in the design of bioprosthetic materials. J. Mater. Sci. Mater. Med.

[64] Bigi A., Cojazzi G., Panzavolta S., Roveri N., Rubini K. (2002). Stabilization of gelatin films by crosslinking with genipin. Biomaterials.

[65] Butler M.F., Ng Y.-F., Pudney P.D.A. (2003). Mechanism and kinetics of the crosslinking reaction between biopolymers containing primary amine groups and genipin. J. Polym. Sci. Part A Polym. Chem.

[66] Bigi A., Cojazzi G., Panzavolta S., Rubini K., Roveri N. (2001). Mechanical and thermal properties of gelatin films at different degrees of glutaraldehyde crosslinking. Biomaterials.

[67] Everaerts F., Torrianni M., Hendriks M., Feijen J. (2007). Quantification of carboxyl groups in carbodiimide cross-linked collagen sponges. J. Biomed. Mater. Res. Part A.

[68] Ulubayram K., Aksu E., Gurhan S.I.D., Serbetci K., Hasirci N. (2002). Cytotoxicity evaluation of gelatin sponges prepared with different cross-linking agents. J. Biomater. Sci. Polym. Ed.

[69] Mimura T., Tabata Y., Amano S. (2011). Transplantation of Corneal Stroma Reconstructed with Gelatin and Multipotent Precursor Cells from Corneal Stroma. Tissue Engineering for Tissue and Organ Regeneration.

[70] Mimura T., Amano S., Yokoo S., Uchida S., Yamagami S., Usui T., Kimura Y., Tabata Y. (2008). Tissue engineering of corneal stroma with rabbit fibroblast precursors and gelatin hydrogels. Mol. Vis.

[71] Lai J.-Y., Li Y.-T. (2010). Evaluation of cross-linked gelatin membranes as delivery carriers for retinal sheets. J. Biomater. Sci. Polym. Ed.

[72] Lai J.-Y., Li Y.-T. (2010). Functional assessment of cross-linked porous gelatin hydrogels for bioengineered cell sheet carriers. Biomacromolecules.

[73] Lai J., Chen K., GH H. (2007). Tissue-engineered human corneal endothelial cell sheet transplantation in a rabbit model using functional biomaterials. Transplantation.

[74] Hsiue G., Lai J., Chen K., Hsu W. (2006). A novel strategy for corneal endothelial reconstruction with a bioengineered cell sheet. Transplantation.

[75] Hsiue G.-H., Lai J.-Y., Lin P.-K. (2002). Absorbable sandwich-like membrane for retinal-sheet transplantation. J. Biomed. Mater. Res.

[76] Vyavahare N.R., Chen W., Joshi R.R., Lee C.-H., Hirsch D., Levy J., Schoen F.J., Levy R.J. (1997). Current progress in anticalcification for bioprosthetic and polymeric heart valves. Cardiovasc. Pathol.

[77] Tsai C.C., Huang R.N., Sung H.W., Liang H.C. (2000). *In vitro* evaluation of the genotoxicity of a naturally occurring crosslinking agent (genipin) for biologic tissue fixation. J. Biomed. Mater. Res.

[78] Grolik M., Szczubiałka K., Wowra B., Dobrowolski D., Orzechowska-Wylęgała B., Wylęgała E., Nowakowska M. (2012). Hydrogel membranes based on genipin-cross-linked chitosan blends for corneal epithelium tissue engineering. J. Mater. Sci. Mater. Med.

[79] Lai J.-Y. (2012). Biocompatibility of genipin and glutaraldehyde cross-linked chitosan materials in the anterior chamber of the eye. Int. J. Mol. Sci.

[80] Khor E. (1997). Methods for the treatment of collagenous tissues for bioprostheses. Biomaterials.

[81] Jorge-Herrero E., Fernández P., Turnay J., Olmo N., Calero P., García R., Freile I., Castillo-Olivares J.L. (1999). Influence of different chemical cross-linking treatments on the properties of bovine pericardium and collagen. Biomaterials.

[82] Kuijpers A., GH E., Krijgsveld J., Zaat S., Dankert J., Feijen J. (2000). Cross-linking and characterisation of gelatin matrices for biomedical applications. J. Biomater Sci Polym Ed.

[83] Yung C.W., Wu L.Q., Tullman J.A., Payne G.F., Bentley W.E., Barbari T.A. (2007). Transglutaminase crosslinked gelatin as a tissue engineering scaffold. J. Biomed. Mater. Res. Part A.

[84] Chen T., Embree H.D., Brown E.M., Taylor M.M., Payne G.F. (2003). Enzyme-catalyzed gel formation of gelatin and chitosan: Potential for *in situ* applications. Biomaterials.

[85] Prasertsung I., Damrongsakkul S., Saito N. (2013). Crosslinking of a gelatin solutions induced by pulsed electrical discharges in solutions. Plasma Process. Polym.

[86] Bhat R., Karim A.A. (2009). Ultraviolet irradiation improves gel strength of fish gelatin. Food Chem.

[87] Brinkman W.T., Nagapudi K., Thomas B.S., Chaikof E.L. (2003). Photo-Cross-Linking of type I collagen gels in the presence of smooth muscle cells: Mechanical properties, cell viability, and function. Biomacromolecules.

[88] Ratanavaraporn J., Rangkupan R., Jeeratawatchai H., Kanokpanont S., Damrongsakkul S. (2010). Influences of physical and chemical crosslinking techniques on electrospun type A and B gelatin fiber mats. Int. J. Biol. Macromol.

[89] Nichol J.W., Koshy S., Bae H., Hwang C.M., Khademhosseini A. (2011). Cell-Laden microengineered gelatin methacrylate hydrogels Jason. Biomaterials.

[90] Pierce B.F., Tronci G., Rössle M., Neffe A.T., Jung F., Lendlein A. (2012). Photocrosslinked co-networks from glycidylmethacrylated gelatin and poly(ethylene glycol) methacrylates. Macromol. Biosci.

[91] Dubruel P., Unger R., Vlierberghe S Van, Cnudde V., Jacobs P.J.S., Schacht E., Kirkpatrick C.J. (2007). Porous gelatin hydrogels: 2. *In vitro* cell interaction study. Biomacromolecules.

[92] Panda P., Ali S., Lo E., Chung B.G., Hatton T.A., Khademhosseini A., Doyle P.S. (2008). Stop-Flow lithography to generate cell-laden microgel particles. Lab Chip.

[93] Chen Y.-C., Lin R.-Z., Qi H., Yang Y., Bae H., Melero-Martin J.M., Khademhosseini A. (2012). Functional human vascular network generated in photocrosslinkable gelatin methacrylate hydrogels. Adv. Funct. Mater.

[94] Nikkhah M., Eshak N., Zorlutuna P., Annabi N., Castello M., Kim K., Dolatshahi-Pirouz A., Edalat F., Bae H., Yang Y. (2012). Directed endothelial cell morphogenesis in micropatterned gelatin methacrylate hydrogels. Biomaterials.

[95] Soman P., Chung P.H., Zhang A.P., Chen S. (2013). Digital microfabrication of user-defined 3D microstructures in cell-laden hydrogels. Biotechnol. Bioeng.

[96] Gauvin R., Chen Y.-C., Lee J.W., Soman P., Zorlutuna P., Nichol J.W., Bae H., Chen S., Khademhosseini A. (2012). Microfabrication of complex porous tissue engineering scaffolds using 3D projection stereolithography. Biomaterials.

[97] Pfizer Gelfilm^®^ absorbable gelatin film.

[98] Pfizer Gelfoam^®^ absorbalbe gelatin compressed sponge.

[99] Qazi Y., Wong G., Monson B., Stringham J., Ambati B.K. (2010). Corneal transparency: Genesis, maintenance and dysfunction. Brain Res. Bull.

[100] Dua H.S., Faraj L.A., Said D.G., Gray T., Lowe J. (2013). Human Corneal Anatomy Redefined: A Novel Pre-Descemet’s Layer (Dua’s Layer). Ophthalmology.

[101] Luo H., Lu Y., Wu T., Zhang M., Zhang Y., Jin Y. (2013). Construction of tissue-engineered cornea composed of amniotic epithelial cells and acellular porcine cornea for treating corneal alkali burn. Biomaterials.

[102] Streilein J.W. (2003). Ocular immune privilege: Therapeutic opportunities from an experiment of nature. Nat. Rev. Immunol.

[103] Lagali N., Griffith M. (2011). Biosynthetic corneas: Prospects for supplementing the human donor cornea supply. Expert Rev. Med. Devices.

[104] Fagerholm P., Griffith M., Lagali N. A Biosynthetic Alternative to Human Donor Tissue.

[105] Allen C.L., Clare G., Stewart E.A., Branch M.J., McIntosh O.D., Dadhwal M., Dua H.S., Hopkinson A. (2013). Augmented dried *versus* cryopreserved amniotic membrane as an ocular surface dressing. PLoS One.

[106] Wilson S., Sidney L., Dunphy S., Rose J.B., Hopkinson A. (2013). Keeping an eye on decellularized corneas: a review of methods, characterization and applications. J. Funct. Biomater.

[107] Lai J.-Y., Chen K.-H., Hsu W.-M., Hsiue G.-H., Lee Y.-H. (2006). Bioengineered human corneal endothelium for transplantation. Arch. Ophthalmol.

[108] Yan J., Qiang L., Gao Y., Cui X., Zhou H., Zhong S., Wang Q., Wang H. (2011). Effect of fiber alignment in electrospun scaffolds on keratocytes and corneal epithelial cells behavior. J. Biomed. Mater. Res. Part A.

[109] Hori K., Sotozono C., Hamuro J., Yamasaki K., Kimura Y., Ozeki M., Tabata Y., Kinoshita S. (2007). Controlled-release of epidermal growth factor from cationized gelatin hydrogel enhances corneal epithelial wound healing. J. Control. Release.

[110] Zorzi G.K., Párraga J.E., Seijo B., Sánchez A. (2011). Hybrid nanoparticle design based on cationized gelatin and the polyanions dextran sulfate and chondroitin sulfate for ocular gene therapy. Macromol. Biosci.

[111] Contreras-Ruiz L., Zorzi G.K., Hileeto D., López-García a, Calonge M., Seijo B., Sánchez a, Diebold Y. (2013). A nanomedicine to treat ocular surface inflammation: Performance on an experimental dry eye murine model. Gene Ther.

[112] Zhang J., Bi R., Hodge W., Yin P., Tse W.H. (2013). A nanocomposite contact lens for the delivery of hydrophilic protein drugs. J. Mater. Chem. B.

[113] Levis H., Daniels J.T. (2009). New technologies in limbal epithelial stem cell transplantation. Curr. Opin. Biotechnol.

[114] Zhu X., Beuerman R.W., Chan-Park M.B.E., Cheng Z., Ang L.P.K., Tan D.T.H. (2006). Enhancement of the mechanical and biological properties of a biomembrane for tissue engineering the ocular surface. Ann. Acad. Med. Singap.

[115] Liu Y., Ren L., Wang Y. (2013). Crosslinked collagen–gelatin–hyaluronic acid biomimetic film for cornea tissue engineering applications. Mater. Sci. Eng. C.

[116] Lu P.-L., Lai J.-Y., Ma D.H.-K., Hsiue G.-H. (2008). Carbodiimide cross-linked hyaluronic acid hydrogels as cell sheet delivery vehicles: Characterization and interaction with corneal endothelial cells. J. Biomater. Sci. Polym. Ed.

[117] Lai J.-Y., Ma D.H.-K., Cheng H.-Y., Sun C.-C., Huang S.-J., Li Y.-T., Hsiue G.-H. (2010). Ocular biocompatibility of carbodiimide cross-linked hyaluronic acid hydrogels for cell sheet delivery carriers. J. Biomater. Sci. Polym. Ed.

[118] Fini M.E. (1999). Keratocyte and fibroblast phenotypes in the repairing cornea. Prog. Retin. Eye Res.

[119] Wang S., Liu W., Han B., Yang L. (2009). Study on a hydroxypropyl chitosan–gelatin based scaffold for corneal stroma tissue engineering. Appl. Surf. Sci.

[120] Beales M.P., Funderburgh J.L., Jester J.V, Hassell J.R. (1999). Proteoglycan synthesis by bovine keratocytes and corneal fibroblasts: Maintenance of the keratocyte phenotype in culture. Invest. Ophthalmol. Vis. Sci.

[121] Gao Y., Jing Y., Cui X.-J., Wang H.Y., Wang Q. (2012). Aligned fibrous scaffold induced aligned growth of corneal stroma cells *in vitro* culture. Chem. Res. Chin. Univ.

[122] Wilson S.L., Wimpenny I., Ahearne M., Rauz S., El Haj A.J., Yang Y. (2012). Chemical and topographical effects on cell differentiation and matrix elasticity in a corneal stromal layer model. Adv. Funct. Mater.

[123] Zhang A.Q., Rubenstein D., Price A.J., Côté E., Levitt M., Sharpen L., Slomovic A. (2013). Evolving surgical techniques of and indications for corneal transplantation in Ontario: 2000–2012. Can. J. Ophthalmol.

[124] Price M.O., Fairchild K.M., Price D.A., Price F.W. (2011). Descemet’s stripping endothelial keratoplasty five-year graft survival and endothelial cell loss. Ophthalmology.

[125] Mimura T., Yamagami S., Amano S. (2013). Corneal endothelial regeneration and tissue engineering. Prog. Retin. Eye Res.

[126] Ishino Y., Sano Y., Nakamura T., Connon C.J., Rigby H., Fullwood N.J., Kinoshita S. (2004). Amniotic membrane as a carrier for cultivated human corneal endothelial cell transplantation. Invest. Ophthalmol. Vis. Sci.

[127] Bok D. (1993). The retinal pigment epithelium: A versatile partner in vision. J. Cell. Sci Suppl.

[128] Bonilha V.L. (2008). Age and disease-related structural changes in the retinal pigment epithelium. Clin. Ophthalmol.

[129] Sparrow J. (2010). The retinal pigment epithelium: In health and disease. Curr. Opin. Mol. Med.

[130] MacLaren R.E., Pearson R.A., MacNeil A., Douglas R.H., Salt T.E., Akimoto M., Swaroop A., Sowden J.C., Ali R.R. (2006). Retinal repair by transplantation of photoreceptor precursors. Nature.

[131] Tomita M., Lavikb E., Klassen H., Zahir T., Langer R., Young M. (2005). Biodegradable polymer composite grafts promote the survival and differentiation of retinal progenitor cells. Stem Cells.

[132] Ballios B.G., Cooke M.J., van der Kooy D., Shoichet M.S. (2010). A hydrogel-based stem cell delivery system to treat retinal degenerative diseases. Biomaterials.

[133] Yaji N., Yamato M., Yang J., Okano T., Hori S. (2009). Transplantation of tissue-engineered retinal pigment epithelial cell sheets in a rabbit model. Biomaterials.

[134] Trese M., Regatieri C.V, Young M.J. (2012). Advances in retinal tissue engineering. Materials.

[135] Del Priore L.V. (2004). Survival of allogeneic porcine retinal pigment epithelial sheets after subretinal transplantation. Invest. Ophthalmol. Vis. Sci.

[136] Margalit E., Fujii G., Lai J., Gupta P., Chen S., Shyu J., Piyathaisere D., Weiland J., de Juan E.J., Humayun M. (2000). Bioadhesives for intraocular use. Retina.

[137] Cohen B., Shefy-Peleg A., Zilberman M. (2014). Novel gelatin/alginate soft tissue adhesives loaded with drugs for pain management: Structure and properties. J. Biomater. Sci. Polym. Ed.

[138] Silverman M.S., Hughes S.E. (1989). Transplantation of photoreceptors to light-damaged retina. Invest. Ophthalmol. Vis. Sci.

[139] Grover C.N., Gwynne J.H., Pugh N., Hamaia S., Farndale R.W., Best S.M., Cameron R.E. (2012). Crosslinking and composition influence the surface properties, mechanical stiffness and cell reactivity of collagen-based films. Acta Biomater.

[140] Schuurman W., Levett P.A., Pot M.W., van Weeren P.R., Dhert W.J. A., Hutmacher D.W., Melchels F.P.W., Klein T.J., Malda J. (2013). Gelatin-methacrylamide hydrogels as potential biomaterials for fabrication of tissue-engineered cartilage constructs. Macromol. Biosci.

[141] Annabi N., Tsang K., Mithieux S.M., Nikkhah M., Ameri A., Khademhosseini A., Weiss A.S. (2013). Highly elastic micropatterned hydrogel for engineering functional cardiac tissue. Adv. Funct. Mater.

[142] Aubin H., Nichol J.W., Hutson C.B., Bae H., Sieminski A.L., Cropek D.M., Akhyari P., Khademhosseini A. (2010). Directed 3D cell alignment and elongation in microengineered hydrogels. Biomaterials.

[143] Keenan T.D.L., Carley F., Yeates D., Jones M.N. A., Rushton S., Goldacre M.J. (2011). Trends in corneal graft surgery in the UK. Br. J. Ophthalmol.

